# Evidence for IL-35 Expression in Diffuse Large B-Cell Lymphoma and Impact on the Patient's Prognosis

**DOI:** 10.3389/fonc.2019.00563

**Published:** 2019-06-28

**Authors:** Frédérique Larousserie, Diakho Kebe, Tony Huynh, Anne Audebourg, Jérôme Tamburini, Benoît Terris, Odile Devergne

**Affiliations:** ^1^Sorbonne Université, INSERM, CNRS, Centre D'Immunologie et des Maladies Infectieuses (Cimi-Paris), Paris, France; ^2^Pathology Department, Cochin Hospital, Assistance Publique-Hôpitaux de Paris, Université Paris Descartes, Paris, France; ^3^Institut Necker Enfants Malades, INSERM, CNRS, Université Paris Descartes, Paris, France; ^4^Hematology Department, Cochin Hospital, Assistance Publique-Hôpitaux de Paris, Université Paris Descartes, Paris, France

**Keywords:** diffuse large B-cell lymphoma, cytokine, interleukin-12 family, prognosis, immune escape

## Abstract

IL-35 is an immunosuppressive cytokine of the IL-12 family consisting of two subunits, EBV-induced gene 3 (EBI3) and p35. It has been shown to play a pro-tumor role in murine tumor models, and in various types of human cancer such as colorectal, pancreatic, or liver carcinoma, its expression has been associated with a worse clinical outcome. Here, we show by analyzing gene expression data from public databases and by immunohistochemical studies that IL-35 is overexpressed by tumor cells in diffuse-large B-cell lymphoma (DLBCL) compared to another type of mature aggressive B-cell lymphoma, Burkitt lymphoma. However, while high IL-35 expression was significantly associated with a worse overall survival in DLBCL patients treated with chemotherapy only (cyclophosphamide, doxorubicin, vincristine, prednisone, CHOP), no significant correlation between IL-35 expression levels and the patient outcome was observed in DLBCL patients treated with CHOP combined to rituximab (R-CHOP), the current conventional treatment. In addition, we found that an anti-IL-35 antibody, clone 15k8D10, used to assess IL-35 expression by immunohistochemistry in various human tissues including tumors does not recognize IL-35 heterodimer, nor its individual subunits EBI3 and p35, but cross-reacts with human IgG1, indicating that IL-35 expression in human cancers needs to be re-evaluated.

## Introduction

Soluble mediators such as cytokines, produced by tumor cells or reactive cells, can contribute to tumor progression and impact on the patient prognosis. Among them there is interleukin (IL)-35, a heterodimeric cytokine of the IL-12 family composed of two non-covalently linked subunits, EBV-induced gene 3 (EBI3) and p35 (1, 2) that regulates immune responses and tumor immunity.

In mice, IL-35 is expressed by specific regulatory T- and B-cell subsets and has been shown to suppress auto-immune and infectious diseases through diverse mechanisms including inhibition of Th17 cell differentiation, suppression of T- or B-cell proliferation, induction of IL-10 production and promotion of regulatory subsets (2–6). Recently, IL-35 was also shown to play a pro-tumor role in murine models of plasmacytoma, melanoma, pancreatic, colon, or breast cancer (7–14). Various mechanisms accounted for IL-35 pro-tumor role: promotion of myeloid cell accumulation in tumors and angiogenesis (7, 13), enhancement of neutrophil infiltration and inflammation (12), facilitation of tumor cell transendothelial extravasation and metastatic colonization (10, 14), and contribution to T cell exhaustion/dysfunction by induction of expression of PD1, TIM3, and LAG3 inhibitory receptors in tumor infiltrating lymphocytes (8).

In humans, the role of IL-35 is less well known and its expression by regulatory T cells is debated (15, 16). However, the high expression of IL-35 at sites of immune escape such as the fetal maternal interface (17) and in tolerogenic dendritic cells (18), favors a key role in immune tolerance. In addition, IL-35 expression by tumor cells or infiltrating lymphocytes has been reported in various types of human tumors including liver, pancreatic, colorectal, or breast cancer (10, 19–25). In most of these studies, IL-35 expression was associated with a worse prognosis (10, 19, 20, 22, 24, 25).

Little is known on the expression of IL-35 in human lymphomas. Previously, we had evidenced that EBI3 is selectively overexpressed by tumor cells in the most frequent type of non-Hodgkin lymphoma among adults, diffuse large B-cell lymphoma (DLBCL), but not in another type of mature aggressive B-cell lymphoma, Burkitt lymphoma (26, 27). DLBCL is heterogeneous with respect to histopathology and clinical features, and by gene expression profiling at least two molecular subtypes have been identified: the germinal center B-cell like (GCB) and the activated B-cell like (ABC) subtypes, the latter being associated with increased aggressiveness and worse clinical outcome. Although the use in recent years of CD20 antibody, rituximab, in combination with the standard chemotherapy treatment, cyclophosphamide, doxorubicin, vincristine, and prednisone (R-CHOP treatment), has improved the survival of DLBCL patients, a fraction of patients is refractory to the treatment or relapses, and new therapeutic approaches are necessary. By analyzing gene expression data available in public database and by immunohistochemical analysis, we had previously shown that EBI3 is expressed by tumor cells in most cases of DLBCL, independently of their GCB or ABC subtypes, with over 30% of tumor cells positive for EBI3 in nearly 80% of DLBCL cases. However, the expression of p35, and of IL-35, was not investigated in these previous studies. Therefore, this study aims at determining whether IL-35 is expressed in DLBCL and constitutes a biomarker for patients with a worse prognosis.

## Methods

### Microarray Analysis

Normalized gene expression values and clinical data from four published microarray studies, GSE4475, GSE4732, GSE10846, and GSE23501, were retrieved from Gene Expression Omnibus. GSE4475 series contained transcriptomic data from 57 Burkitt lymphoma patients and 142 DLBCL patients analyzed on U133A gene chip (28). GSE4732 series included 54 Burkitt lymphoma and 219 DLBCL analyzed on LymphDx 2.7k GeneChip (29). Of these, 24 cases of Burkitt lymphoma and 40 cases of DLBCL were also analyzed with Affymetrix U133 Plus 2.0 microarrays. This series comprises primary mediastinal DLBCL that were excluded from our analysis. GSE10846 (30) and GSE23501 (31) comprise data from 412 and 69 DLBCL patients, respectively, analyzed on Affymetrix U133 Plus 2.0 microarrays. As mentioned in the original publications describing these publicly available gene profiling datasets, these studies were conducted according to protocols approved by local ethics commission [Charité University Hospital, Berlin; National Cancer Institute Institutional Review Board, United States, (28–31)].

Based on *EBI3* and *p35* gene expression, DLBCL cases were classified into “IL-35-high” and “IL-35-low” cases. Because most DLBCL cases have high expression of *EBI3, p35* expression constitutes the limiting factor for IL-35 expression. Therefore, cases were first classified into “*p35*-low” or “*p35*-high” based on whether *p35* expression level was below or above the median. Cases classified as “*p35*-low,” as well as the few “*p35*-high” cases that displayed low levels of *EBI3* expression (arbitrarily, we chose the same median value) were considered as “*IL-35*-low,” while all others were classified as “*IL-35*-high.”

### Immunohistochemistry

Human tissues analyzed in this study were retrieved from the files of the Department of Pathology of Cochin Hospital (Paris) and included six cases of non-neoplastic reactive lymph nodes, one case of hepatocellular carcinoma, and 75 cases of B-cell lymphomas collected between 2004 and 2014 and classified as DLBCL according to the criteria of World Health Organization. DLBCL cases were selected because clinical data such as age-adjusted international prognosis index (aaIPI) and/or progression free survival were available. Immunohistochemical studies, performed retrospectively on fixed tissues collected for diagnosis purpose, were conducted in accordance with the declaration of Helsinki and article L.1121–1 of French law. The use of clinical and pathologic records was in agreement with French laws and ethical guidelines related to the protection of the patient.

Before staining, serial tissue sections were deparaffinized, rehydrated, subjected to antigen retrieval by heat pre-treatment using citrate buffer, and incubated with a peroxide-methanol buffer to quench endogenous peroxidase activity. Mouse anti-IL-35/EBI3 monoclonal antibody (mAb) (Imgenex, clone 15k8D10) was used at 4 μg/ml, and mouse 2G4H6 anti-EBI3 mAb (17) was used at 3 μg/ml. 2G4H6 anti-EBI3 Ab was raised against recombinant bacterial EBI3 and specifically detects recombinant or natural human EBI3 by ELISA, immunoprecipitation, western blot or immunohistochemical analysis (17). p35 was detected using goat polyclonal antibody (C19, Santa Cruz Biotechnology, 3 μg/ml). In some cases, expression of p35 by tumor cells was verified by using rabbit anti-p35 mAb (Ab131039, Abcam, 5 μg/ml). Binding of primary antibodies was detected by an indirect avidin-biotin peroxidase technique and DAB as chromogen. Sections were counterstained with Mayer hematoxylin. Images were captured on a NanoZoomer 2.0-RS slide scanner (Hamamatsu Corporation) and processed with NDP Viewer. For scoring of IL-35 expression in DLBCL, tissue sections were first tested for EBI3 and, when positive (≥30% positive tumor cells), also tested for p35 on serial sections. The identity of positive cells in immunohistochemical staining was established by pathologists based on standard histopathological criteria and according to WHO classification of tumors of haematopoietic and lymphoid tissues. Cases with ≥30% tumor cells positive for both EBI3 and p35 were classified as “IL-35-positive,” while all other cases were classified as “IL-35-negative.”

### Western Blot Analysis

The specificity of anti-IL-35 or anti-p35 Abs used in this study was tested against various recombinant proteins, including IL-12 (R&D Systems), EBI3 (EBI3-Flag protein purified in the lab using anti-Flag M2 beads from the supernatant from transfected COS7 cells), IL-35 (non-covalently linked heterodimer produced in HEK293 cells, Peprotech), IL-35-Fc (fusion protein produced in HEK293 cells, consisting of EBI3 linked to p35 fused to the Fc portion of human IgG1, Enzo Life Science), control IgG1-Fc protein produced in CHO cells (Enzo Life Science), as well as natural IgG1 (purified from human serum, Sigma), and the cell lysate of a DLBCL cell line, OCI-Ly3 cell (gift from Laura Pasqualucci, Columbia University). Purified proteins or cell lysate were subjected to SDS-PAGE under reducing conditions and transferred to nitrocellulose for immunoblotting. Blots were incubated with mouse anti-EBI3 2G4H6 mAb (17), mouse anti-IL-35/EBI3 mAb (Imgenex, clone 15k8D10), goat anti-p35 Ab (C19, Santa Cruz Biotechnology), rabbit anti-p35 mAb (Ab131039, Abcam), or goat anti-IL-12 polyclonal Ab (R&D Systems), followed by HRP-conjugated secondary Abs (GE Healthcare). Peroxidase reaction was developed with chemiluminescence reagents (Thermo Fisher Scientific).

### Statistical Analysis

Data were analyzed using Prism 7 software. Student's *t*-test or log rank test for Kaplan-Meier survival curve were used. A *p* < 0.05 was considered as significant.

## Results

### IL-35 Is Overexpressed in DLBCL

**Figure 1:**
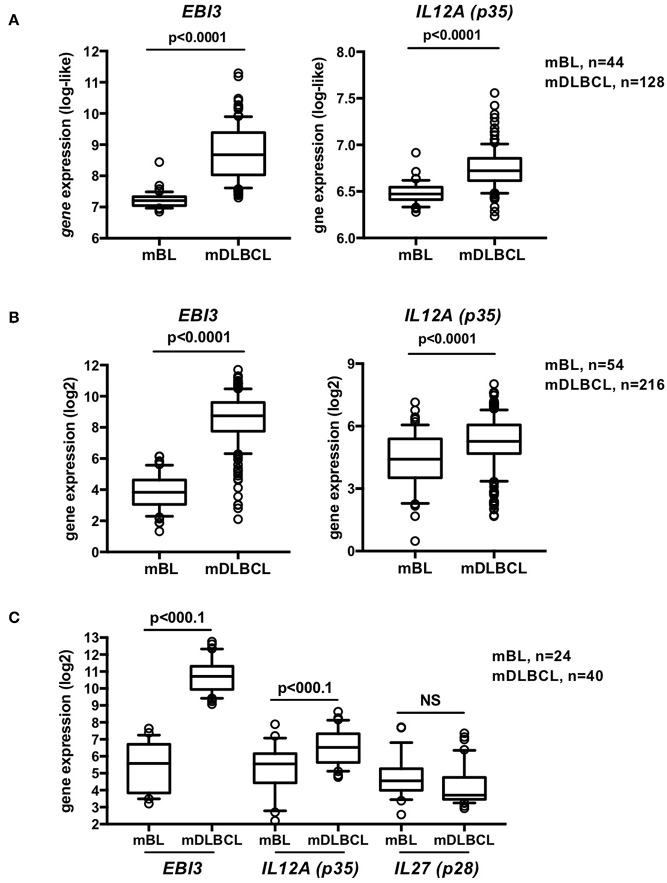
IL-35 is overexpressed in DLBCL compared to BL. **(A–C)** Gene expression values for *EBI3* and *p35* (*IL12A*) from B-cell lymphomas classified by molecular profiling as Burkitt lymphoma (mBL) or DLBCL (mDLBCL) were extracted from GSE4475 **(A)** and GSE4732 **(B)**. While the microaarrays used in these studies do not contain probes for *p28* gene, a subgroup of samples from the GSE4732 study was analyzed using U133 Plus 2.0 array that contains a probe for *p28*, and gene expression of *EBI3, p35* and *p28* (*IL27*) for this subset of samples is shown in **(C)**. Data (normalized signal intensity) are represented as box plot (10–90% percentile) and outliers are shown. *p*-values of *t*-tests are indicated.

We first investigated whether, similar to *EBI3, p35* is selectively overexpressed in DLBCL compared to Burkitt lymphoma. Analysis of the GSE447 and GSE4732 microarray datasets that we previously analyzed for *EBI3* expression (27) and totalized 98 cases of Burkitt lymphoma and 344 cases of DLCLC defined by molecular gene profiling, showed that not only *EBI3* but also *p35* levels were significantly upregulated in DLBCL compared to Burkitt lymphoma (Figures 1A,B). In contrast, detection of *p28* (*IL27*), the alternative partner for EBI3 to form IL-27 (EBI3/p28 heterodimer), resulted in low signals that were not increased in DLBCL compared to Burkitt lymphoma (Figure 1C), which was consistent with our previous lack of p28 detection by immunohistochemistry in tumor cells of DLBCL (26). These results indicate that IL-35, but not IL-27, is specifically overexpressed in DLBCL.

### Lack of Specificity of Anti-IL-35 15k8D10 mAb

To investigate which cell types express IL-35 in DLBCL, we tested different commercial Abs for IL-35 detection by immunohistochemistry. In several published immunohistochemical studies of IL-35 expression in human cancers including hepatocellular carcinomas, colorectal cancer or breast cancer, the expression of IL-35 heterodimer was investigated by using a commercial mouse anti-IL-35 mAb (clone 15k8D10, original supplier Imgenex) (7, 20–22, 24, 32). This mAb, raised against a fusion protein formed of human EBI3 and p35 subunits fused to the Fc domain of human IgG1, was initially described as specific for IL-35 and later indicated to be specific for EBI3 subunit by immunoblot or immunohistochemical analyses of paraffin-embedded human tissues.

**Figure 2:**
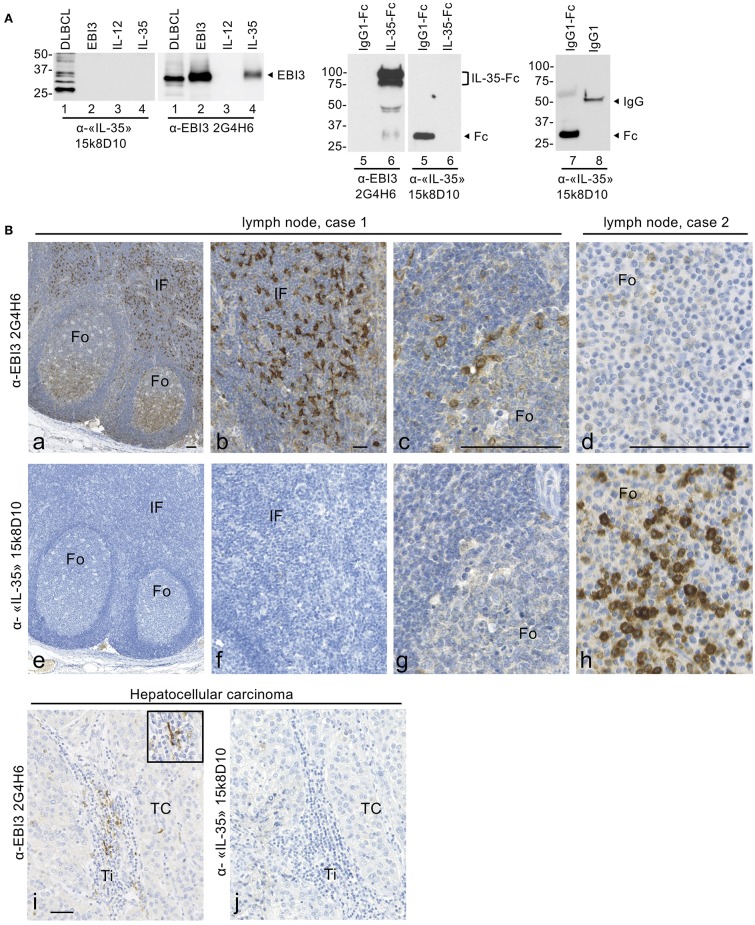
15k8D10 anti-IL-35/EBI3 mAb lacks specificity for EBI3. **(A)** The reactivity of 15k8D10 anti-IL-35/EBI3 mAb was tested by immunoblot against the cell lysate of OCI-Ly3 DLBCL cell line (30 μg total protein extract) and various recombinant proteins, as indicated (50 ng in lanes 2–7, and 100 ng in lane 8). Recombinant IL-12 (p35/p40 heterodimer) was used as a source of p35. The 2G4H6 anti-EBI3 mAb was used in parallel, as a positive control. The position of molecular weight standards is indicated on the left (in kDa). Because of post-translational modification, intracellular EBI3 is detected as a 33-kDa protein (lane 1) and secreted EBI3 as a 34-kDa protein (lanes 2 and 4). The positions of IgG1-Fc (28 kDa), IL-35-Fc (a doublet around 85 kDa), and IgG1 (50 kDa) are indicated. **(B)** Immunohistochemical analysis with 15k8D10 anti-IL-35/EBI3 mAb. Serial sections of non-neoplastic lymph nodes (a–h) or of hepatocellular carcinoma (i,j) were stained with 15k8D10 anti-IL-35/EBI3 mAb or anti-EBI3 2G4H6 mAb as indicated. Fo, follicle; IF, interfollicular area; TC, tumor cells; Ti, Tumor infiltrates. In (i), the inset shows a larger magnification of tumor infiltrating EBI3-positive cells with a morphology consistent with dendritic cells. The bar represents 50 μm.

Surprisingly, when we tested anti-IL-35/EBI3 15k8D10 mAb by western blotting against various recombinant proteins or cell lysate, in parallel to a previously characterized specific mouse anti-EBI3 mAb [2G4H6 clone, (17)], it failed to detect recombinant IL-35 produced as a non-covalently linked heterodimer in 293 cells (Figure 2A, lane 4) or EBI3 and p35 subunits individually (Figure 2A, lanes 2, 3), and yielded several non-specific cross-reacting bands in the cell lysate from OCI-Ly3 DLBCL cell line (Figure 2A, lane 1). It also did not detect a recombinant IL-35-IgG1-Fc fusion protein produced in 293 cells (lane 6), but, unexpectedly, recognized an IgG1-Fc portion produced in CHO cells (Figure 2A, lanes 5, 7) as well as natural human IgG1purified from human serum (Figure 2A, lane 8).

We further tested the reactivity of anti-IL-35/EBI3 15k8D10 mAb in immunohistochemical analyses of formalin-fixed paraffin-embedded tissues, in parallel to 2G4H6 EBI3 mAb. EBI3 is expressed at high levels by activated dendritic cells and B-cells (26, 33), and in previous immunohistochemical analyses of EBI3 expression in human reactive lymph nodes, we observed high expression of EBI3 by a subset of germinal center B cells located in the light zone of B-cell follicles that corresponds to proliferating activated centrocytes, as well as by cells with extended cytoplasmic processes located in interfollicular areas identified as dendritic cells (26, 34). In human tumors, strong expression of EBI3 by infiltrating dendritic cells was also observed (35, 36). When we stained serial sections of non-neoplastic reactive lymph nodes (*n* = 6) and of a case of hepatocellular carcinoma with 2G4H6 anti-EBI3 mAb or 15k8D10 mAb, we observed a discordant pattern of staining between both mAbs (Figure 2B). Cells labeled with 2G4H6 mAb in germinal centers and previously identified as activated centrocytes (26) (Figures 2Ba,c) were not recognized by 15k8D10 mAb (Figures 2Be,g). Similarly, EBI3-positive cells in interfollicular areas or tumor infiltrates (Figures 2Bb,i) were not recognized by 15k8D10 mAb (Figures 2Bf,j). In addition, in two cases of reactive lymph nodes that had been fixed in formalin/acetic acid instead of neutral formalin, we observed a strong staining of lymphoid cells within B-cell follicles with 15k8D10 mAb, that was not observed with 2G4H6 mAb (Figures 2Bd,h).

Thus, from both western blotting or immunohistochemical analyses, we conclude that 15k8D10 mAb does not allow the specific detection of IL-35, and that it reacts with the Fc portion of human IgG1 (and possibly IgG1-positive B-cells) under specific conditions.

### Heterogenous Expression of IL-35 by Tumor Cells in DLBCL

**Figure 3:**
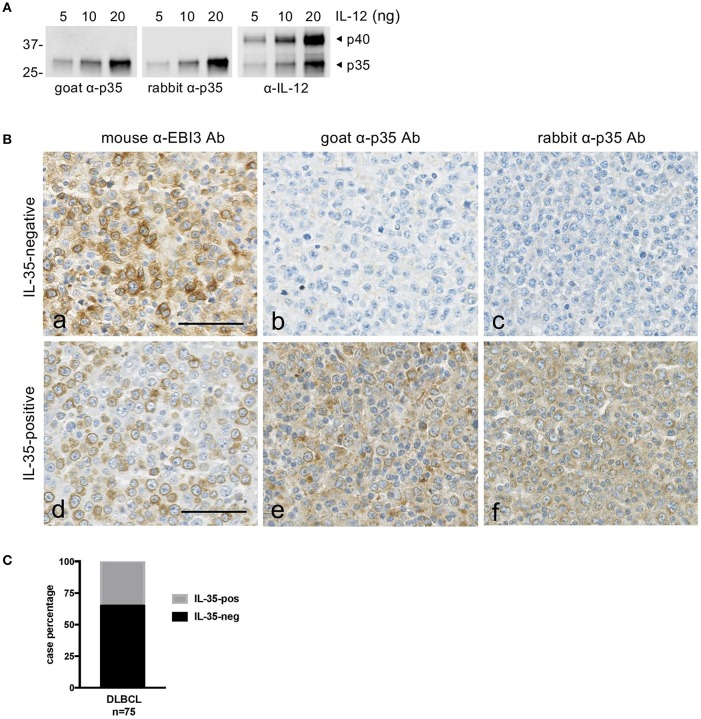
Expression of IL-35 by tumor cells in DLBCL. **(A)** Specificity of the anti-p35 antibodies used for immunohistochemical studies. Goat or rabbit polyclonal anti-p35 (α-p35) antibodies were tested by western blot using the indicated amount of recombinant IL-12. Anti-IL-12 (α-IL-12) antibody was used as a positive control to detect p35 and p40 subunits. The position of molecular weight standards is indicated on the left (in kDa). **(B)** Serial sections of DLBCL tissues were stained with anti-EBI3 or anti-p35 antibodies as indicated. Representative cases classified as “IL-35-negative” and “IL-35-positive” are shown. The bar represents 50 μm. **(C)** Graph indicating the percentage of IL-35-negative and -positive cases among the DLBCL tested by immunohistochemistry.

Because there is no Ab to specifically detect IL-35 heterodimer, we analyzed IL-35 expression in DLBCL tissues (*n* = 75) by staining serial tissue sections with 2G4H6 mouse anti-EBI3 mAb and with goat polyclonal anti-p35 Abs or rabbit anti-p35 mAb (Figure 3). Anti-p35 Abs were verified to specifically detect p35 by western blot (Figure 3A). In line with microarray data, we observed that expression of EBI3 by tumor cells was associated with variable levels of p35 expression by these cells, ranging from undetectable expression to moderate expression by most tumor cells (Figure 3B). Of the 75 DLBCL cases tested, 26 (35%) had ≥30% tumor cells positive for both EBI3 and p35 and were scored as IL-35-positive (Figure 3C). In some cases, cells other than tumor cells, such as infiltrating leukocytes (possibly macrophages or plasma cells based on their morphology) were also positive for EBI3 and p35.

### Correlation Between IL-35 Expression and Prognosis of DLBCL Patients

Next, we investigated whether IL-35 expression correlates or not with an adverse prognosis in DLBCL patients.

**Figure 4:**
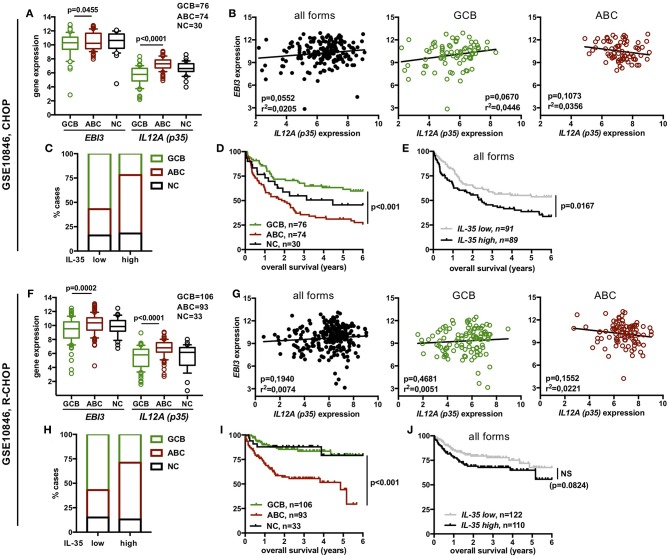
Expression of IL-35 among the different molecular forms of DLBCL and correlation with overall survival in DLBCL patients from the GSE10846 dataset. Data are shown for CHOP- **(A–E)** or R-CHOP **(F–J)**-treated patients. **(A,F)** Gene expression of *EBI3* and *p35* among the various molecular forms of DLBCL defined by gene profiling (log2 scale). Box plot limits indicate the 10–90 percentile of normalized intensity values, and outliers are shown. *p*-values of student *t*-tests are indicated. **(B,G)** Correlation between *EBI3* and *p35* expression levels (log2 scale) among all molecular forms (GCB + ABC + NC) or within GCB or ABC forms. R-square and *p*-value (Pearson correlation test) are indicated. **(C,H)** Distribution of ABC, GCB and NC cases among “*IL-35*-low” and “*IL-35*-high” DLBCL cases. **(D–I,E–J)** Percentage of overall survival (y-axis) over time (x-axis) (Kaplan-Meier survival curve) of DLBCL patients classified by molecular subtypes **(D,I)** or by IL-35 expression levels **(E,J)**. *p*-values of log-rank test are indicated. NS, not significant.

**Figure 5:**
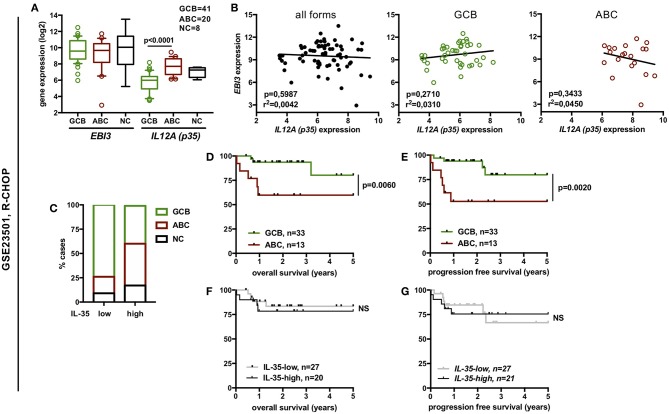
Expression of IL-35 among the different molecular forms of DLBCL and correlation with overall or progression free survival of the patients from the GSE23501 dataset. **(A)** Gene expression of *EBI3* and *p35* among the various molecular forms of DLBCL defined by gene profiling (log2 scale). Box plot limits indicate the 10–90 percentile of normalized intensity values, and outliers are shown. *p*-values of student *t*-tests are indicated. **(B)** Correlation between *EBI3* and *p35* expression levels (log2 scale) among all molecular forms (GCB + ABC + NC) or within GCB or ABC forms. R-square and *p*-value (Pearson correlation test) are indicated. **(C)** Distribution of ABC, GCB, and NC cases among “*IL-35*-low” and “*IL-35*-high” DLBCL cases. **(D–G)** Percentage (y-axis) of overall survival or progression-free survival over time (x-axis) (Kaplan-Meier survival curve) of DLBCL patients classified by molecular subtypes **(D,E)** or by IL-35 expression levels **(F,G)**. *p*-values of log-rank test are indicated. NS, not significant.

In the two princeps transcriptomic studies analyzed above for IL-35 expression, DLBCL patients were treated with CHOP or CHOP-like regimen only. Therefore, to search for a possible correlation between IL-35 expression level and clinical outcome of DLBCL patients treated with the current standard treatment, i.e., R-CHOP, we analyzed two other microarray datasets for IL-35 expression, GSE10846 (Figure 4) and GSE23501 (Figure 5). Among publicly available transcriptomic datasets, the GSE10846 dataset was chosen because it comprises a large cohort of patients treated with R-CHOP (*n* = 232) for which information about the overall survival of the patient was available (30). It also contains a substantial series of patients treated with CHOP or similar regimens (*n* = 180, in part common to the previously studied GSE4732 series) allowing comparison between CHOP- and R-CHOP-treated patients. The GSE23501 dataset contains transcriptomic data from 69 DLBCL patients including 47 R-CHOP-treated patients with available clinical information (overall survival and progression free survival) (31). In all series, DLBCL cases had been classified by gene profiling into GCB and ABC forms or left “not classified” (“NC”).

We observed that expression level of *p35* and, for GSE10846 series also of *EBI3*, was significantly higher in ABC forms compared to GCB forms (Figures 4A,F, 5A). However, in each series, no significant correlation between *EBI3* and *p35* expression levels was observed, neither when analyzing all molecular forms together, nor when analyzing GBC or ABC forms separately (Figures 4B,G, 5B).

**Figure 6:**
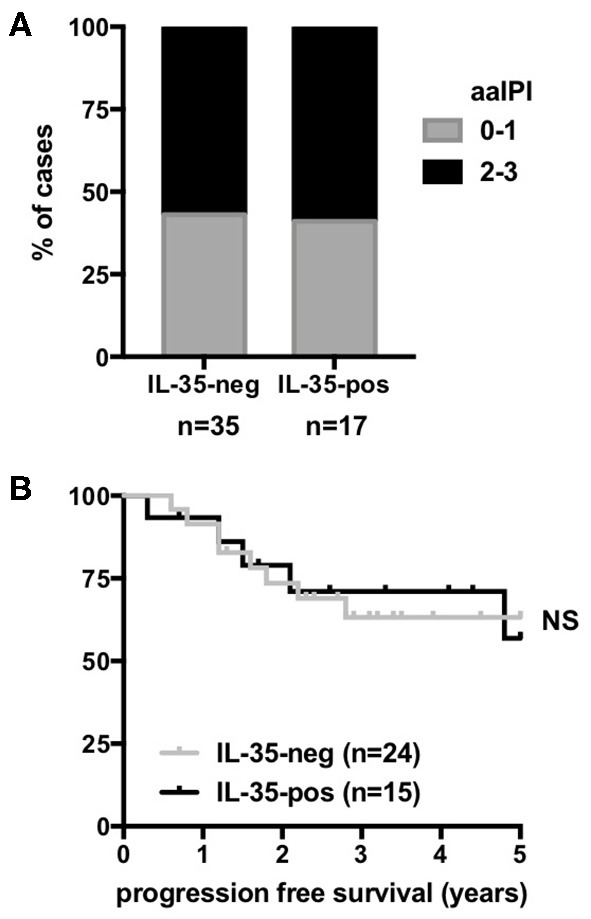
Correlation between IL-35 expression assessed by immunohistochemistry and the outcome of DLBCL patients. Distribution of age-adjusted international prognosis index (aa IPI) scores **(A)** and percentage (y-axis) of progression-free survival over time (x-axis) (Kaplan-Meier survival curves) of R-CHOP-treated DLBCL patients **(B)** classified into “IL-35-positive” and “IL-35-negative” by immunohistochemistry.

To evaluate whether levels of IL-35 expression impact the patient prognosis, we classified DLBCL cases into IL-35 “high” or “low” cases. IL-12 family cytokines are heterodimers composed of an α and β subunit the biosynthesis of which requires the coordinated expression of the α and β subunits within producing cells. Although p35 (α-subunit) is expressed by a broader spectrum of cell types than its cognate β subunit (p40 or EBI3), it is usually expressed at lower levels that the β subunit in cells co-expressing both subunits and was previously shown to be the limiting partner for the expression of the α/β heterodimer (37). Thus, in DLBCL, most of which have high expression of *EBI3, p35* is likely to be the limiting factor for IL-35 expression. Therefore, to classify DLBCL cases into “IL-35-low” and “IL-35-high” cases, cases were first classified into “*p35*-low” or “*p35*-high” based on whether *p35* expression level was below or above the median. Cases classified as “*p35*-low,” as well as the rare “*p35*-high” cases that displayed low levels of *EBI3* expression were considered as “*IL-35*-low,” while all others were classified as “*IL-35*-high.” As expected from the higher expression of IL-35 subunits in ABC over GCB forms, we observed an enrichment of ABC forms among “IL-35-high” patients (Figures 4C,H, 5C). In each cohort, these ABC forms were of worse clinical outcome, as shown by shorter overall survival and progression-free survival (Figures 4D,I, 5D,E). However, although “IL-35-high” cases from CHOP- or R-CHOP-treated patients of the GSE10846 dataset were enriched in ABC forms in similar proportions (60 and 58% ABC forms among “IL-35 high” patients in CHOP and R-CHOP-treated patients, respectively), the overall survival of “IL-35-high” cases was statistically lower than that of “IL-35-low” cases only among CHOP-treated patients, but not among R-CHOP treated patients (Figures 4E,J). In line with these data, neither the overall survival nor the progression free survival of R-CHOP treated patients from the GSE23501 cohort showed statistical difference between “IL-35-low” or “IL-35-high” patients (Figures 5F,G). In addition, in our series of patients analyzed for IL-35 by immunohistochemistry, no correlation between positivity for IL-35 expression (defined as <30% tumor cells positive for both EBI3 and p35) and the age adjusted international prognosis index (available for 52 cases, Figure 6A) or the progression free survival of R-CHOP treated patients (*n* = 39, Figure 6B) was observed. Thus, the addition of rituximab to CHOP regimen in DLBCL patients appears to circumvent the positive correlation between high IL-35 expression and a worse clinical outcome.

**Figure 7:**
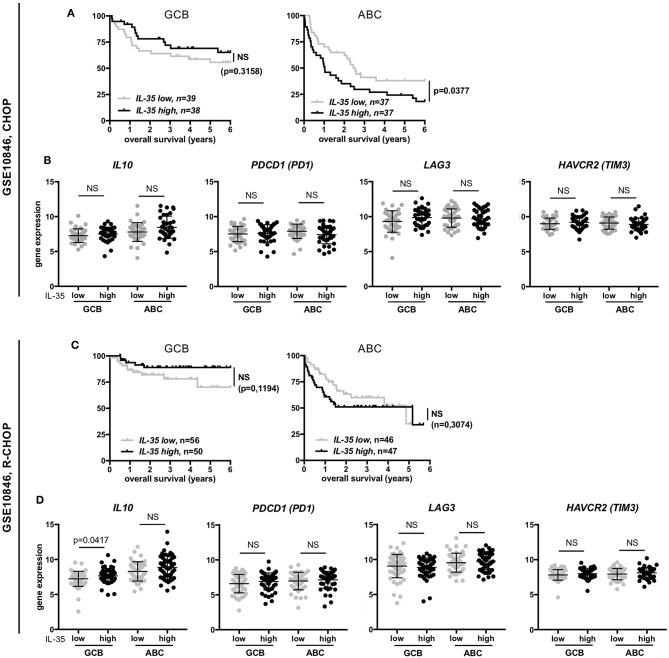
Correlation among GCB or ABC molecular forms of DLBCL between IL-35 expression and either overall survival or expression of IL-10 and co-inhibitory receptors. **(A,C)** Percentage (y-axis) of overall survival over time (x-axis) (Kaplan-Meier survival curve) of CHOP- or R-CHOP-treated GCB or ABC DLBCL patients from the indicated series, associated with low or high IL-35 expression levels. *p*-values of log-rank test are indicated. **(B,D)** Gene expression of the indicated genes among the various molecular forms of DLBCL defined by gene profiling (log2 scale) is shown. Horizontal line indicates the mean (±SD). *p*-values of student *t*-tests are indicated. NS, not significant.

In the GSE10846 series that contains a large number of patients, we further evaluated the impact of IL-35 expression on the patient prognosis within a given molecular form (Figure 7). Among GCB forms, the overall survival was not statistically different between “IL-35-high” and “IL-35-low” cases, whether patients were treated with CHOP or R-CHOP (Figures 7A,C). Among ABC forms, “IL-35-high” patients had a shorter overall survival than “IL-35-low” patients when treated with CHOP (Figure 7A), but not when treated with R-CHOP (Figure 7C). Thus, in R-CHOP treated DLBCL patients, no significant correlation between IL-35 expression and the patient outcome could be observed, whether analyzing all subtypes together, or GCB and ABC forms separately.

Last, because IL-35 has been involved in induction of IL-10 and of the co-inhibitory molecules PD1, TIM3, and LAG3 (8), that are known to be expressed in DLBCL and can impact the patient outcome (38–41), we investigated whether increased expression of IL-35 was associated with increased expression of IL-10, PD1 (*PDCD1*), TIM3 (*HAVCR2*), or LAG3 in these patients (Figures 7B,D). Except for IL-10 whose expression was higher in “IL-35-high” patients of the GCB subtype from the R-CHOP cohort (but not in the equivalent patients from the CHOP cohort), we did not observe higher expression of these molecules in “IL-35-high” patients compared to “IL-35-low” patients. Specifically, the shorter overall survival of “IL-35-high” patients of ABC forms from the CHOP-treated cohort was not associated with increased expression of IL-10, PD1, LAG3, or TIM3 genes in these patients (Figure 7B).

## Discussion

In previous studies, we had shown that EBI3 is expressed by tumor cells in various types of B- or T-cell lymphomas, especially in DLBCL, in most cases in the absence of p28, its binding partner to form IL-27 (26, 36). This observation led us to investigate whether the other partner of EBI3 to form IL-35, p35, was expressed in DLBCL and whether IL-35 expression constitutes a prognosis marker for DLBCL patients. We show here that IL-35 expression is detected in DLBCL at increased levels compared to Burkitt lymphoma, and is highest among DLBCL of ABC form that are of worse prognosis. However, while data from transcriptomic studies indicates that IL-35 expression is associated with a shorter overall survival in CHOP-treated patients, this association is lost among R-CHOP-treated patients. In line with these findings, analysis of IL-35 expression by immunohistochemistry in R-CHOP treated patients showed no tendency for a worse outcome of patients with higher expression of IL-35. Thus, in R-CHOP patients whose clinical outcome is significantly improved compared to CHOP-treated patients, tumor expression of IL-35 does not appear as a key factor driving tumor progression.

In mouse models, the immunosuppressive or anti-tumor roles of IL-35 have been linked to its ability to induce the expression of IL-10 and the immune checkpoints LAG3, TIM3, and PD1 in T cells, leading to T cell exhaustion and decreased anti-tumor immunity (8). In the DLBCL cases analyzed here, we did not observe a positive correlation between expression of IL-35 and that of IL-10, LAG3, PD1, or TIM3. Thus, other factors or mechanisms, such as genetic alterations, are likely to predominate for driving the expression of these molecules in DLBCL (42).

In addition, our study shows that the 15k8D10 anti-IL-35/EBI3 mAb used to detect IL-35 expression in many published studies is not specific. It does not detect EBI3 nor p35, and reacts with human IgG1 and other unknown proteins in immunoblot analysis, and may detect IgG1-positive B lymphocytes as well as irrelevant proteins in immunohistochemical studies. In a sneaky way, its immunoreactivity in immunohistochemical assays appears to vary depending on the conditions of tissue fixation, and its reactivity for human IgG1 proteins as tested in immunoblot analyses was variable depending on the protein source (human serum, recombinant IgG1 protein expressed in CHO or 293 cells). IgG are subjected to post-translational modifications that vary depending on the cellular source and may affect their immunogenicity (43, 44), which could account for the variable detection of IgG1 proteins observed with 15k8D10 mAb.

The lack of specificity of 15k8D10 anti-IL-35/EBI3 mAb challenges previous studies performed with this antibody. For example, while two studies concluded from immunohistochemistry with 15k8D10 mAb that tumor cells express IL-35 in hepatocellular carcinoma (20, 21), we did not detect EBI3 expression by most tumor cells in this type of tumor (13 cases analyzed, Figure 2B and data not shown).

The lack of significant correlation between IL-35 expression and prognosis in DLBCL patients contrasts with previous findings in other human tumors for which IL-35 expression in tumor was associated with a worse prognosis. However, whereas most studies identified IL-35 as a factor positively linked to tumor progression (10, 19, 20, 22, 24, 25), others suggested that IL-35 may play an anti-tumor role (21, 23). The impact of IL-35 in cancer most likely results from a combination of various factors involving expression of IL-35 receptor, tumor genomics, and specific features of tumor microenvironment. Of note, because in several of the studies mentioned above, IL-35 expression was detected using 15k8D10 mAb, some of the conclusions from these studies need to be confirmed.

Altogether, our results do not point for a major role of IL-35 in tumor progression in R-CHOP treated DLBCL patients. They also suggest that IL-35 expression in different human cancers, including hepatocellular carcinoma, colorectal carcinomas, and breast cancer, and its correlation with the patient's prognosis need to be re-evaluated.

## Data Availability

All datasets generated for this study are included in the manuscript.

## Ethics Statement

We analyzed gene expression data from published microarray datasets retrieved from Gene Expression Omnibus. As mentioned in the original publications describing these publicly available gene profiling datasets, the studies were conducted according to protocols approved by local ethics commission [Charité University Hospital, Berlin; National Cancer Institute Institutional Review Board, United States, (28–31)].

Immunohistochemical studies, performed retrospectively on fixed tissues collected for diagnosis purpose, were conducted in accordance with the declaration of Helsinki and article L.1121–1 of French law. The use of clinical and pathologic records was in agreement with French laws and ethical guidelines related to the protection of the patient.

## Author Contributions

OD and FL designed the study and analyzed data. OD, DK, and AA performed and analyzed experiments. TH and JT collected clinical data. BT contributed tissue samples. OD wrote the manuscript. All authors reviewed the manuscript.

### Conflict of Interest Statement

OD is an inventor of a patent on IL-35. The remaining authors declare that the research was conducted in the absence of any commercial or financial relationships that could be construed as a potential conflict of interest.
